# Birth weight and body mass index z-score in childhood brain tumors: A cross-sectional study

**DOI:** 10.1038/s41598-018-19924-8

**Published:** 2018-01-26

**Authors:** Kuan-Wen Wang, Russell J. de Souza, Adam Fleming, Donna L. Johnston, Shayna M. Zelcer, Shahrad Rod Rassekh, Sarah Burrow, Lehana Thabane, M. Constantine Samaan

**Affiliations:** 1 Department of Pediatrics, McMaster University, Hamilton, Ontario Canada; 2 Division of Pediatric Endocrinology, McMaster Children’s Hospital, Hamilton, Ontario Canada; 3 Medical Sciences Graduate Program, McMaster University, Hamilton, Ontario Canada; 4 Department of Health Research Methods, Evidence and Impact, McMaster University, Hamilton, Ontario Canada; 5 Division of Pediatric Hematology/Oncology, McMaster Children’s Hospital, Hamilton, Ontario Canada; 6 Division of Pediatric Hematology/Oncology, Children’s Hospital of Eastern Ontario, Ottawa, Ontario Canada; 7 Pediatric Hematology Oncology, Children’s Hospital, London Health Sciences Center, London, Ontario Canada; 8 Division of Pediatric Hematology/Oncology/BMT, Department of Pediatrics, British Columbia’s Children’s Hospital, Vancouver, BC Canada; 9 Division of Orthopedic Surgery, Department of Surgery, McMaster University Medical Centre, Hamilton, Ontario Canada; 10 Department of Anesthesia, McMaster University, Hamilton, Ontario Canada; 11 Centre for Evaluation of Medicines, St. Joseph’s Health Care, Hamilton, Ontario Canada; 12 Biostatistics Unit, St Joseph’s Healthcare-Hamilton, Hamilton, Ontario Canada

## Abstract

Children with brain tumors (CBT) are at higher risk of cardiovascular disease and type 2 diabetes compared to the general population, in which birth weight is a risk factor for these diseases. However, this is not known in CBT. The primary aim of this study was to explore the association between birth weight and body mass measures in CBT, compared to non-cancer controls. This is a secondary data analysis using cross-sectional data from the CanDECIDE study (n = 78 CBT and n = 133 non-cancer controls). Age, sex, and birth weight (grams) were self-reported, and confirmed through examination of the medical records. Body mass index (BMI) was calculated from height and weight measures and reported as kg/m^2^. BMI z-scores were obtained for subjects under the age of 20 years. Multivariable linear regression was used to evaluate the relationship between birth weight and BMI and BMI z-score, adjusted for age, sex, puberty, and fat mass percentage. Higher birth weight was associated with higher BMI and BMI z-score among CBT and controls. In conclusion, birth weight is a risk factor for higher body mass during childhood in CBT, and this may help the identification of children at risk of future obesity and cardiometabolic risk.

## Introduction

Brain tumors are the most common cause of cancer-related deaths in children^1,2^. While brain tumors are a heterogeneous group with some fairly aggressive subtypes, advancements in imaging and therapeutic breakthroughs have increased the number of children surviving these tumors^1,2^. This important milestone has been offset by the emergence of co-morbidities and premature mortality in survivors^3–7^. While traditionally reported outcome determinants include tumor recurrence and secondary tumors, recent evidence suggests that children with brain tumors (CBT) are at higher risk of premature cardiovascular diseases including hypertension, cerebrovascular events and type 2 diabetes compared to non-cancer controls^8–10^. While the mechanisms leading to these cardiometabolic disorders are not well understood, the combined burden of the tumor and treatment with these emerging chronic disorders will increasingly contribute to adverse outcomes in CBT as these survivors live longer and get older.

The global obesity epidemic is the main catalyst of cardiometabolic disorders in the general population^11–14^, and the association of obesity with future type 2 diabetes and cardiovascular diseases has been tracked to childhood^15^. Defining the determinants of obesity, cardiovascular disease and diabetes in CBT will permit the prioritization of children who need early intervention to improve survivors’ quality of life and lifespan.

Over the past two decades, evidence has validated the role of birth weight as a risk factor for adult obesity and cardiometabolic disorders^16,17^; however, it is unclear if birth weight in CBT is a risk factor for obesity during childhood. Given that CBT have excess adiposity in comparison to non-cancer controls^18^, it may be a potential early predictor of further adverse cardiometabolic outcomes. In addition, while CBT have similar rates of overweight/obesity when compared to non-cancer controls, this still leaves one-in-four CBT in the overweight/obese category based on body mass measures^19^. Body mass index (BMI) is the most used screening tool to assess obesity risk, and may still be a predictor of future cardiometabolic risk, as these children get older. Therefore, the primary aim of this paper was to explore if birth weight is a risk factor for higher body mass in CBT, compared to non-cancer controls.

## Results

### Population characteristics

We included 78 CBT (n = 33 females [42.3%]) and 133 non-cancer controls (n = 60 females, [45.1%]) in this study. The characteristics of the study population are shown in Table 1. The two groups have similar age, sex, ethnicity, BMI, BMI z-score, and birth weight distribution. However, the control group was taller and weighed more than CBT. On the other hand, CBT had higher fat mass percentage (%FM) (CBT 25.80 ± 9.60% versus controls 22.40 ± 9.80%, p = 0.01).

**Table 1:** Characteristics of study population.

Variables	Control	CBT	p-value
	Total (n = 133) Mean ± SD	Total (n = 78) Mean ± SD	
Age at enrollment (years)	14.10 ± 2.70	15.10 ± 7.30	0.64
Sex, No. (%)			0.69
Female	60 (45.10)	33 (42.30)	
Male	73 (54.90)	45 (57.70)	
Ethnicity, No. (%)			0.26
European	91 (68.40)	59 (75.60)	
Others	42 (31.60)	19 (24.40)	
Puberty, No. (%)			0.001
Pre-pubertal	17 (12.80)	24 (30.80)	
Pubertal	116 (87.20)	54 (69.20)	
Height (cm)	162.70 ± 15.00	151.00 ± 25.20	<0.001
Weight (kg)	60.00 ± 21.40	53.40 ± 24.90	0.007
BMI (kg/m^2^)	21.70 ± 4.20	21.40 ± 4.70	0.49
BMI z-score	0.49 ± 1.10	0.47 ± 1.10	0.92
BMI category, No. (%)			0.62
BMI%ile < 85 or BMI < 25	88 (66.20)	49 (62.80)	
BMI%ile ≥ 85 or BMI ≥ 25	45 (33.80)	29 (37.20)	
Fat mass percentage (%)	22.40 ± 9.80	25.80 ± 9.60	0.01
Birth weight (g)	3491.30 ± 487.40	3436.60 ± 516.50	0.43
Pregnancy gestation (weeks)	38.90 ± 2.30	39.90 ± 1.80	0.001

Abbreviations: CBT, Children with Brain Tumors; SD, Standard Deviation; BMI, Body Mass Index.

Participants born at full-term comprised the largest groups (CBT n = 45 [57.70%]; controls n = 65 [48.90%]). More controls were born pre-term (CBT n = 3 [3.80%]; controls n = 21 [15.80%], p = 0.008) while both have similar distribution for early-term (CBT n = 10 [12.80%]; controls n = 24 [18.00%], p = 0.32) and late-term (CBT n = 20 [25.60%]; controls n = 23 [17.30%], p = 0.15).

The majority of both CBT and controls were born appropriate for gestational age (AGA) (CBT n = 60 [76.90%]; controls n = 90 [67.70%]). Both groups had a similar distribution for those who were born small for gestational age (SGA) (CBT n = 11 [14.10%]; controls n = 12 [9.00%], p = 0.25) while large for gestational age (LGA) was more common in controls (CBT n = 7 [9.00%]; controls n = 31 [23.30%], p = 0.009). Maternal gestational diabetes was reported in two CBT (2.60%) and in one control (0.80%), while preeclampsia was reported in five CBT (6.40%) and 10 controls (7.50%).

The majority of the subjects had normal birth weight (CBT n = 62 [79.50%]; controls n = 104 [78.20%]). Six CBT (7.70%) and six controls (4.50%) were born with a low birth weight (<2500 grams). High birth weight (>4000 grams) was reported in ten CBT (12.80%) and 23 controls (17.30%).

### Tumor characteristics and treatments

Brain tumor characteristics and therapeutic modalities are reported in Table 2. The most common tumors in this study population were low-grade gliomas (n = 45 [57.70%]). Brain tumors were equally distributed between supratentorial and infratentorial regions. The treatments used in participants were surgery alone (n = 25 [32.00%]), and a combination of surgery, radiotherapy, and chemotherapy (n = 24 [30.80%]). Other single treatment options included radiotherapy alone (n = 2 [2.60%]), and chemotherapy alone (n = 7 [9.00%]). Other combinations of treatment modalities were surgery and radiotherapy (n = 6 [7.70%]), surgery and chemotherapy (n = 5 [6.40%]), and radiotherapy and chemotherapy (n = 1 [1.30%]). Eight CBT (10.20%) were managed conservatively at the time of inclusion in the study.

**Table 2:** Brain tumor type, location, and treatments (n = 78).

**Variables**	**No. (%)**
**Brain tumor type**	
Non-NF-1, low grade glioma	34 (43.60)
PNET/Medulloblastoma	17 (21.80)
NF-1, low grade glioma	11 (14.10)
CNS germ cell tumors	6 (7.70)
Subependymal giant cell astrocytoma	3 (3.80)
Ependymoma	2 (2.60)
Craniopharyngioma	2 (2.60)
Meningioma	1 (1.30)
Atypical teratoid/rhabdoid tumor	1 (1.30)
Choroid plexus papilloma	1 (1.30)
**Brain tumor location**	
Supratentorial	36 (46.20)
Infratentorial	42 (53.80)
**Brain tumor treatments**	
Surgery	60 (76.90)
Radiotherapy	33 (42.30)
Chemotherapy	37 (47.40)
No treatment	8 (10.30)

Abbreviations: CNS, Central Nervous System; PNET, Primitive Neuroectodermal Tumor; NF-1, Neurofibromatosis Type 1.

### The association of birth weight and body mass

To explore the association of birth weight with body mass, we performed multivariable regression analyses in CBT, adjusted for age, sex, puberty, and %FM. For every unit increase in birth weight, BMI increased by 0.18 units (95%CI 0.03,0.33; p = 0.02) in CBT and by 0.17 units (95%CI 0.07,0.27; p = 0.001) in controls. Similarly, for every unit increase in birth weight, BMI z-score also increased by 3.69 units (95%CI 1.12,6.25; p = 0.006) in CBT and by 2.15 units (95%CI 0.75,3.55; p = 0.003) in controls.

To determine if the association between birth weight and body mass differs between CBT and controls, an interaction term (birth weight*brain tumor status) was introduced (Table 3). Birth weight is associated with body mass, and the effect of birth weight on body mass was similar between CBT and controls (BMI β = 0.02; 95% CI −0.15, 0.20; p = 0.80; BMI z-score β = 2.02; 95% CI −0.62,4.67; p = 0.13).

**Table 3:** Interaction analysis of body mass measures and birth weight in CBT and non-cancer controls.

BMI				
Variables	Main Effect		Interaction	
	Estimated β (95% CI)	p-value	Estimated β (95% CI)	p-value
Birth weight	0.18 (0.09,0.27)	<0.001	0.17 (0.06,0.28)	0.003
Brain tumor status	−0.03 (−0.04, −0.01)	<0.001	−0.11 (−0.73,0.51)	0.73
Interaction^a^	—	—	0.02 (−0.15,0.20)	0.80
**BMI z-score**				
Birth weight	2.81 (1.54,4.09)	<0.001	2.10 (0.52,3.68)	0.009
Brain tumor status	−0.33 (−0.51, −0.14)	0.001	−7.48 (−16.83,1.86)	0.12
Interaction^a^	—	—	2.02 (−0.62,4.67)	0.13

Abbreviations: CBT, Children with Brain Tumors; BMI, Body Mass Index; CI, Confidence Interval. Models were adjusted for age, sex, puberty, fat mass percentage.

^a^The interaction term was birth weight*brain tumors status (yes/no).

## Discussion

The emergence of cardiovascular diseases and type 2 diabetes in survivors of childhood brain tumors are likely to contribute to adverse prognoses, and there is an urgent need to identify the drivers of these outcomes to mitigate their effects on the life span and quality of life. In this study, we demonstrate that birth weight is a risk factor for higher body mass in CBT during childhood, and this relationship was similar to that noted in non-cancer controls.

The influence of the in- and ex-utero environments on the risk of obesity and cardiometabolic risk is an important determinant of health outcomes, based on evidence from studies in the general population^20–22^. One of the potential early and feasible measures that forecast these outcomes is birth weight.

Birth weight is driven by several factors, including genes that determine body size and growth, and the intrauterine environment^23^. It is estimated that 10–40% of birth weight is driven by genetic factors, with several loci identified to suggest genetic links with body weight and mass^23,24^. In addition, fetal metabolic programing *in utero* in response to the intrauterine environment contributes to cardiometabolic health postnatally through epigenetic and other mechanisms^25^.

The exposure of an embryo to an adverse intrauterine environment and excess metabolic stress leads to the re-programming of the metabolic pathways, to adapt to in-utero scarcity or excess of nutrients^22,26^. Clinically, this manifests with infants being born small or large for gestational age^27^. However, it is likely that at intermediate stages of metabolic stress, some babies may have a birth weight within the normal range, but have been exposed to an environment that can alter their metabolic trajectory^28^.

The evidence for the association of certain categories of birth weight with adult BMI and cardiometabolic disorders was highlighted in studies from David Barker and the Dutch famine cohort and others, and showed that birth weight and maternal-fetal undernutrition was linked to low birth weight that was associated with adult obesity and adverse cardiometabolic outcomes^29–34^.

In addition, the link between birth weight and obesity was highlighted in previous reports showing that those born SGA or LGA to be at risk of adult obesity^22^. However, recent studies report that in those with a birth weight that is sometimes within the normal range, or who have high birth weight (>4000 grams), are at risk of adult obesity^28,35,36^. Contrary to previous evidence^37–39^, some studies did not show linear, J-shaped or U-shaped associations of birth weight with adult obesity^35,36^.

Our data show a positive relationship between birth weight and body mass measures in CBT and controls in childhood. This is congruent with recent large-scale studies that have provided further evidence of similar results in the general pediatric population^27,40,41^. Birth weight may help identify those CBT who are at risk of adult obesity. Detailed study of growth paths and longer follow-up period are needed to determine if birth weight is a risk factor for obesity in CBT as they reach adulthood.

While available evidence suggest that obese children are at risk of becoming obese adults^42–44^, the association between birth weight, childhood BMI and future cardiometabolic risk is more complex^45^. It has been reported that birth weight below 3.4 kg, which is still considered appropriate for gestational age, and high BMI during childhood were independently associated with increased risk of coronary heart disease^20^. The association between low birth weight and type 2 diabetes was also reported^46,47^. The evidence indicates that both birth weight and BMI need to be scrutinized in CBT and controls to identify subjects who are at an increased risk of cardiometabolic disorders, as they appear to be independently linked to these outcomes.

While the majority of evidence has focused on high (>4000 g) and low (<2500 g) birth weight and its association with obesity risk^28,35^, it is less clear how a normal birth weight affect this trajectory of adult obesity and cardiometabolic outcomes in CBT. Our data suggest that as weight trends higher while still within the normal range, this represents a risk factor for higher body mass. However, more prospective data sets are required to validate this observation and its association with cardiometabolic outcomes in CBT.

One of the strengths of our study is the inclusion of non-cancer controls to provide a comparison group. It is a strength to have this control group because when all is equal for birth weight between cases and controls, we can test the effect of having cancer and therapy on the association between birth weight and BMI.

It has been shown that CBT have increased adiposity early in life post completion of therapy, and are at higher risk of cardiovascular diseases and diabetes compared to the general population, despite having similar BMI to controls^9,18,48^. CBT can have disproportionate effects of their tumor and its treatment on cardiometabolic outcomes at a similar obesity rate, and birth weight may be a potential risk factor for these outcomes^18,49,50^.

There are several limitations in this study. We did not have sufficient power to determine the association of birth weight with young adult BMI, as the number of young adult subjects in our study was small. While we demonstrate that birth weight is positively associated with body mass measures in adolescence, our data does not distinguish whether this is a result of the expansion of lean body mass or fat mass, as BMI is a measure of total body mass. A recent study showed that birth weight was associated with fat-free mass, but not with fat mass among children and adolescents^51^. This will require further clarification in future studies.

This analysis is cross-sectional and therefore it is not clear if subjects were overweight or obese before their brain tumor diagnoses. In addition, the data used in the study were collected as a part of the Canadian Study of Determinants of Endometabolic Health in Children (CanDECIDE study)^52^, and this paper is not related to the primary study question. Therefore, the study was not designed to answer this question specifically. Prospective collection of data from diagnosis onwards and correlating growth data with those from earlier time points may help define growth patterns of those survivors at risk of obesity.

In conclusion, cardiometabolic disorders are occurring at a relatively young age in CBT, and are emerging as significant morbidities and as potential determinants of longevity^5,9,48^. Our results suggest that birth weight is a risk factor for higher body mass in CBT in the early years post treatment. Future studies need to focus on determining the early origins of obesity and cardiometabolic risk in survivors. This will help identify survivors who are at particular risk of these complications, and birth weight may be one of the risk markers used to stratify cardiometabolic risk in CBT.

## Methods

### Participants

This secondary data analysis used data that were collected as a part of the CanDECIDE Study. This is a cohort study conducted at McMaster Children’s Hospital in Hamilton, Ontario, Canada^52,53^.

Briefly, we recruited participants who were 5 years and older, with no history of autoimmune diseases or infection, and have not received immunosuppressive therapy for at least 15 days prior to enrollment. Participants were consecutively recruited and the study recruitment took place between November 2012-March 2017. The Hamilton Integrated Research Ethics Board approved the study, and participants provided written informed consent. The study procedures were carried out in accordance with relevant guidelines and legal regulations.

### Clinical Data and Anthropometric measures

The collected data included age, sex, ethnicity, puberty, pregnancy gestation, maternal gestational diabetes and preeclampsia, and reported birth weight using standardized questionnaires^52,53^. While reported birth weight correlates with measured birth weight^54^, the reported birth weight was verified from the medical records. In CBT, we also collected data regarding tumor type, location, sidedness and treatment modalities.

Gestation was defined for those born at less than 37 weeks as preterm, between 37–38 + 6/40 weeks as early term, 39–40 + 6/40 weeks as full term, and 41–41 + 6/40 weeks as late term gestations^55^. Normal birth weight was defined to be between 2500–4000 grams^56^. Infants born SGA were defined as those with a birth weight below the10th percentile, AGA as 10th-90th percentile, and LGA as above 90th percentile using Canadian reference ranges^57^.

Anthropometric measurements performed included height measured to 0.1 cm using a stadiometer, and weight measured using an electronic weighing scale (Seca, USA) and measured to the closest 0.1 kg. BMI was calculated in kg/m^2^ for all subjects. For those under 20 years of age (CBT n = 62, controls n = 133), BMI percentile and BMI z-scores were also obtained based on the Children’s BMI Tool for Schools^58^ and the Centers for Disease Control and Prevention (CDC) growth chart^59^, respectively. Subjects with BMI ≥ 85^th^ –<95^th^ percentile were classified as overweight, and those above 95^th^ percentile were classified as obese^60^.

Adiposity was determined by measuring fat mass percentage (%FM) using the Tanita body fat monitor (Tanita Corporation, Illinois, USA) for those under 18 years of age, and with the InBody520 body composition analyzer (Biospace Co., Ltd, Korea) for those 18 years and older as previously reported^18^.

### Statistical Analysis

All analyses were performed using PASW version 18 statistical package^61^. Kolmogorov-Smirnov test was used to assess the normality of data distribution, and data were log-transformed if they were non-normally distributed. Outliers were examined with box plot and visual inspection of extreme values. Multiple imputations was done for missing data^62^. Mean and standard deviation (SD) were reported for continuous variables, while the categorical variables were reported as counts with percentages. Independent sample t-tests were used to compare continuous variables between CBT and non-cancer controls while chi-square tests were performed to compare categorical variables.

To explore the association between birth weight and body mass measures in CBT, multivariable linear regression analysis was performed in this group. The dependent variables included BMI and BMI z-scores in separate models. The independent variables included birth weight, age, sex, puberty, and %FM.

The relationship between birth weight and body mass in CBT and non-cancer controls was explored by adding an interaction term (birth weight*brain tumor status). Both CBT and non-cancer controls were included in the regression analysis.

Results were presented as estimated β coefficients, 95% confidence intervals (CI), and associated p-value. The criterion for statistical significance was set at alpha = 0.05.

### Data availability

The dataset used for statistical analysis for the current study is available from the corresponding author.
